# Anomalous transport phenomena in Weyl metal beyond the Drude model for Landauʼs Fermi liquids

**DOI:** 10.1088/1468-6996/15/6/064401

**Published:** 2014-11-25

**Authors:** Ki-Seok Kim, Heon-Jung Kim, M Sasaki, j-f Wang, L Li

**Affiliations:** 1 Department of Physics, POSTECH, Pohang, Gyeongbuk 790-784, Korea; 2 Institute of Edge of Theoretical Science (IES), Hogil Kim Memorial building 5th floor, POSTECH, Pohang, Gyeongbuk 790-784, Korea; 3 Department of Physics, College of Natural Science, Daegu University, Gyeongbuk 712-714, Korea; 4 Department of Physics, Faculty of Science, Yamagata University, Kojirakawa, Yamagata 990-8560, Japan; 5 Wuhan National High Magnetic Field Center (WHMFC), Huazhong University of Science and Technology, Wuhan 430074, People’s Republic of China

**Keywords:** Weyl metal, Berry curvature, chiral anomaly, axion electrodynamics, topological Drude model, topological Fermi-liquid theory

## Abstract

Landauʼs Fermi-liquid theory is the standard model for metals, characterized by the existence of electron quasiparticles near a Fermi surface as long as Landauʼs interaction parameters lie below critical values for instabilities. Recently this fundamental paradigm has been challenged by the physics of strong spin–orbit coupling, although the concept of electron quasiparticles remains valid near the Fermi surface, where Landauʼs Fermi-liquid theory fails to describe the electromagnetic properties of this novel metallic state, referred to as Weyl metal. A novel ingredient is that such a Fermi surface encloses a Weyl point with definite chirality, referred to as a chiral Fermi surface, which can arise from breaking of either time reversal or inversion symmetry in systems with strong spin–orbit coupling, responsible for both the Berry curvature and the chiral anomaly. As a result, electromagnetic properties of the Weyl metallic state are described not by conventional Maxwell equations but by axion electrodynamics, where Maxwell equations are modified with a topological-in-origin spatially modulated 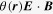 term.

This novel metallic state was realized recently in Bi
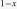
Sb_*x*_ around 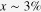 under magnetic fields, where the Dirac spectrum appears around the critical point between the normal semiconducting (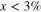) and topological semiconducting phases (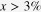) and the time reversal symmetry breaking perturbation causes the Dirac point to split into a pair of Weyl points along the direction of the applied magnetic field for a very strong spin–orbit coupled system.

In this review article, we discuss how the topological structure of both the Berry curvature and the chiral anomaly (axion electrodynamics) gives rise to anomalous transport phenomena in 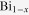
Sb_*x*_ around 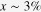 under magnetic fields, thus modifying the Drude model of Landauʼs Fermi liquids.

## 1. Introduction

The Drude model is the first theory that we learn even in graduate courses in solid-state physics [1]. Although we have no idea when we first learn the theory, we become surprised later at the fact that such a simple point of view can be borne out in extremely complex organized structures of solids. Learning Blochʼs theorem [1], we start to understand essentially why the free electron theory works. However, this is just the beginning of a series of surprises in realizing the power of the Drude model because the Drude model still works well even if electron correlations become strong enough to enhance the band mass of an electron much larger than that of weakly correlated metals. This deep question is resolved within Landauʼs Fermi-liquid theory, the backbone of which is the existence of electron quasiparticles, where the strongly correlated metallic state is connected to a degenerate Fermi-gas state adiabatically even if the weight of the energy state and the effective band mass near the Fermi surface become quite renormalized [2].

Recently this common belief has been challenged by the discovery of a novel metallic state, referred to as Weyl metal [3–5]. Even if the concept of electron quasiparticles remains valid, Landauʼs Fermi-liquid theory fails to explain anomalous transport properties of this new metal phase. First of all, the characteristic feature of Weyl metal originates from its band structure. Assume the band structure of a topological insulator, described by an effective Dirac Hamiltonian in momentum space [6]:

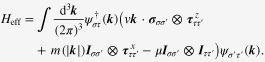
Here 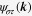 represents a four-component Dirac spinor, where *σ* and *τ* are spin and chiral indexes, respectively. 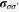 and 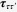 are Pauli matrices acting on spin and ‘orbital’ spaces. The relativistic dispersion is represented in the chiral basis, where each eigenvalue of 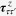 expresses either + or − chirality, respectively. The mass term can be formulated as 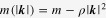, where 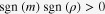 corresponds to a topological insulating state whereas 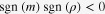 corresponds to a normal band insulating phase. *μ* is the chemical potential, controlled by doping. One may regard that this simplified effective model can be derived from a realistic band structure in Bi
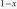
Sb_*x*_, describing dynamics of electrons near the 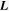 point in momentum space.

**Figure 1.**
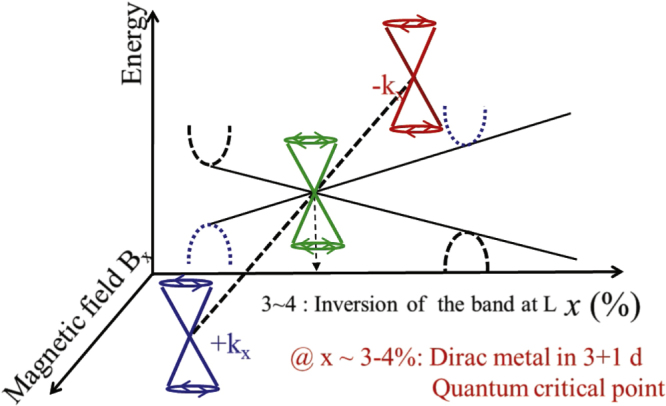
A schematic phase diagram of Bi
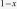
Sb_*x*_ with a schematic band structure near the 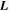 point. A band inversion occurs around 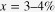, giving rise to a gapless Dirac spectrum near the 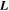 point. Applying magnetic fields to this Dirac metal phase, the Dirac band splits into a pair of Weyl bands with definite chirality along the direction of the magnetic field, analogous to the polarization of a monopole and antimonopole pair induced by the magnetic field. (Figure from Kim *et al* [20].)

It has been demonstrated that the mass gap can be tuned to vanish at 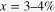 in Bi
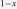
Sb_*x*_, allowing us to reach the critical point between the topological and band insulating phases [7–9]. It is straightforward to show that this gapless Dirac spectrum splits into a pair of Weyl points, thus breaking time reversal symmetry—for example, applying magnetic fields in the gapless semi-conductor

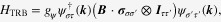
where 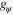 is the Landé g-factor. The band touching point 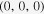 of the Dirac spectrum shifts into 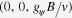 and 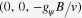 for each chirality along the direction of the magnetic field, given by

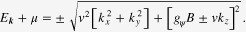
Now each spectrum is described by a two-component Weyl spinor with definite chirality, referred to as Weyl metal. See figure 1. One can also find this type of spectrum breaking inversion symmetry instead of time reversal symmetry [4, 5, 10].

An important feature of Weyl metal results from the existence of the Berry curvature on each Fermi surface [3], referred to as a chiral Fermi surface because each Fermi surface encloses a Weyl point with definite chirality. When an electron moves on a chiral Fermi surface adiabatically, it feels an internal force given by the Berry curvature [11, 12]. Considering that the Weyl Hamiltonian is given by 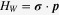 with momentum 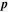 and spin 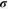 near one Weyl point with + chirality, the existence of the Berry curvature is rather analogous to the dynamics of a spin under an adiabatic change of an external magnetic field 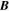 described by 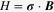 [13]. Recall that each Weyl point can be identified by a magnetic monopole in momentum space, regarded to be the source of the Berry curvature [11, 12]. When both + and − magnetic charges exist at the same momentum point, there will be no net magnetic charge and no Berry curvature. Applying magnetic fields, each ± magnetic charge becomes ‘polarized’ in momentum space. However, unexpected and more interesting physics result beyond the presence of the Berry curvature on the single chiral Fermi surface. First of all, the dynamics of electron quasiparticles in one chiral Fermi surface are not free from those in the other paired chiral Fermi surface [14]. This anomalous relation between the pair of Weyl points can be understood easily, considering one- dimensional problems. At first, the dynamics of electrons near a 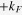 point appear independent in relation to those near a 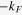 point, where *k*_*F*_ is a Fermi momentum. However, they cannot be independent because there is a band structure to connect their dynamics. As a result, a decrease in the number of − chiral electrons must give rise to an increase in that of + chiral electrons, and vice versa [1]. Generally speaking, although symmetries or associated currents are conserved at the classical level, they are not respected at the quantum level sometimes; this is referred to as anomaly [15]. Here chiral currents are not conserved quantum mechanically; thus the anomaly is called chiral anomaly [16]. This chiral anomaly also arises in three-space dimensions when the band structure shows a pair of chiral Fermi surfaces (Weyl points), i.e., in Weyl metal [14].

A cautious person may point out that the band structure of Weyl metal is essentially the same as that of graphene, where the + chirality Weyl spectrum at the 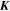 point and the − chirality Weyl spectrum at the 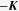 point allow us to call graphene two-dimensional Weyl metal [17]. However, there is one critical difference between Weyl metal and graphene. Weyl electrons in the pair of Weyl points are not independent in Weyl metal as just discussed, whereas they have ‘nothing’ to do with each other in graphene. It is true that Weyl points in graphene can be regarded as a pair of Weyl points with opposite chirality according to the no-go theorem of Nielsen and Ninomiya [18, 19]. In addition, they can be shifted and merged into one Dirac point, applying effective ‘magnetic’ fields to couple with the pseudo-spin of graphene [4]. However, such an anomaly relation does not exist between the pair of Weyl points in graphene, which means that currents are conserved separately for each Weyl cone in contrast with the Weyl metal case as long as inter-valley scattering can be neglected. A crucial distinguishing aspect between two and three dimensions is that the irreducible representation of the Lorentz group is a four-component Dirac spinor in three dimensions, whereas it is a two-component Weyl spinor in two dimensions [15]. As a result, the pair of Weyl points originates from the Dirac point in three dimensions, where such a pair of Weyl points is ‘connected’ through the Dirac sea. On the other hand, each Weyl point of the pair exists ‘independently’ in two dimensions. Chiral anomaly is the key feature of Weyl metal.

The foregoing discussion defines our problem clearly. How should we modify the Drude model of Landauʼs Fermi-liquid theory to incorporate the topological information of both the Berry curvature and the chiral anomaly? How can we generalize Landauʼs Fermi-liquid theory to encode the topological structure? In section 2, we discuss how the Drude model becomes generalized for a pair of chiral Fermi surfaces, based on [20] and [21]. This section covers an introduction to topological properties of the Weyl metallic state within the Boltzmann transport theory. In this context we suggest that readers who are not very familiar with the chiral anomaly check out this part carefully in combination with the Introduction. In section 3 we interpret recent experiments on Bi
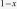
Sb_*x*_ [20], based on the Boltzmann transport theory with the modified Drude model, discussed in section 2. Readers familiar with Weyl metal may focus on this section, skipping section 2. In section 4 we discuss a topological Fermi-liquid theory, reviewing the Weyl Lagrangian in the first quantization, which gives rise to the generalization of the Drude model. In particular, we justify our effective field theory for a pair of chiral Fermi surfaces, reproducing the enhancement of the longitudinal magneto-electrical conductivity given by the Boltzmann transport theory. Because this section is devoted to an aspect of quantum field theory, readers who are not particularly interested in the theoretical aspect may skip this section and proceed to the last section. Considering that this review article focuses on the introduction to topological properties of the Weyl metallic state in a pedagogical way as much as possible, we suggest referring to a recent preprint on the topological Fermi-liquid theory [22] for rigorous derivations from a microscopic theory. In the last section, we discuss a possible direction of this research, pointing out interesting problems with Weyl metal, with sections 5.2 and 5.3 based on [22] and [23], respectively. In the appendix, we discuss the role of the topological-in-origin 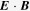 term in the Weyl metallic phase. In particular, we prove that the angle coefficient 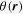 is given by 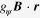, where 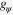 is the Landé g-factor and 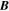 is an external magnetic field. This tells us that electromagnetic properties of the Weyl metallic state are described not by conventional Maxwell equations but by axion electrodynamics [24].

## 2. How to describe a Weyl metallic state I: Boltzmann transport theory

### 2.1. The Boltzmann transport theory with the Drude model in Landau’s Fermi-liquid state

The Boltzmann transport theory combined with the Drude model is our basic framework for metals described by Landauʼs Fermi-liquid theory [25]. The Boltzmann equation is given by

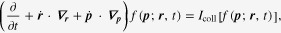
where 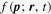 is the distribution function with the conjugate momentum 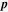 of the relative coordinate and the center of mass coordinate (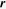, *t*) in the Wigner transformation of the lesser Green function [26]. Based on the semi-classical wave-packet picture, one may regard that 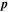 is the momentum to characterize an internal state and 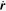 is the group velocity of the particle [11, 12]. The dynamics of the internal momentum and the center of mass coordinate are described by the Drude model,

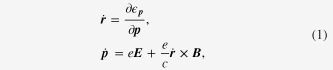
and correspond to the group velocity with band dispersion 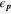 and the equation of motion with the Lorentz force, respectively [1]. The right-hand side in the Boltzmann equation contains various collision terms, including electron correlations and impurity-scattering effects. In this review article, we do not take into account electron correlations.

Incorporating the Drude model into the Boltzmann equation with impurity scattering, we obtain

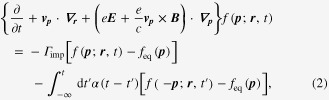
where 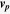 is the group velocity and 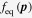 is the equilibrium distribution function. The collision part consists of two types of impurity-scattering contributions [27]. The first is an elastic impurity-scattering term in the relaxation-time approximation, where 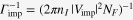 with an impurity concentration *n*_*I*_, its potential strength is *V*_imp_, and the density of states *N*_*F*_ corresponds to the mean free time, the time scale between events of impurity scattering. The second is a weak localization (weak antilocalization) term, expressed in a non-local way, which originates from multiple impurity scattering. 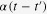 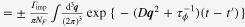 may be regarded as the diffusion kernel, which becomes more familiar, performing the Fourier transformation as follows:

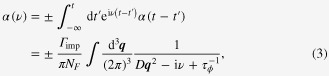
where the sign + (−) represents the weak localization (weak antilocalization). *D* is the diffusion coefficient, and *N*_*F*_ is the density of states at the Fermi energy. This expression is supplemented by the upper cut off in the momentum integral, given by the reciprocal of the mean free path 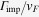 with the Fermi velocity *v*_*F*_, and 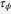 corresponds to the lower cutoff, identified by the phase-coherence lifetime.

It is straightforward to solve this equation in the linear-response regime. Then, the electrical conductivity can be found from the following expression:

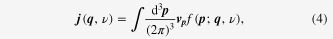
where 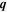 and *ν* are the momentum and frequency for the center of mass coordinate.

### 2.2. A Boltzmann transport theory with a ‘topological’ Drude model

The Weyl metallic state consists of a pair of chiral Fermi surfaces. As a result, the ‘minimal’ model for Boltzmann equations should be constructed for each Fermi surface, given by

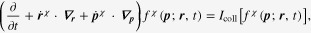
where the superscript of *χ* represents each Weyl point with either + or − chirality.

An essential idea is to introduce the topological structure of both the Berry curvature and the chiral anomaly into the Boltzmann equation framework [28], modifying the Drude model in an appropriate way for Weyl metal [11, 12]. We call it ‘topological’ Drude model, given by

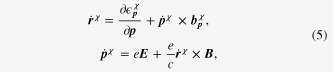
where 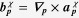 is the Berry curvature and 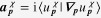 is the Berry connection with Blochʼs eigen state 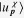. The emergence of the anomalous velocity term is a key feature in the topological Drude model, originating from the Berry curvature on the chiral Fermi surface. The Berry magnetic field is determined by

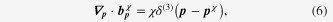
where the ‘magnetic’ charge 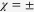 of a monopole in momentum space is given by the chirality of the Weyl point appearing at 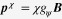 as discussed in the Introduction.

It is straightforward to find the solution to these semi-classical equations of motion, given by [28]

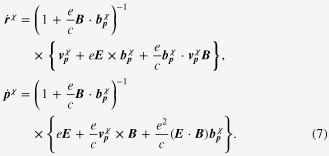
Here 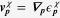 is the group velocity with a band structure 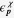. We would like to point out that this band structure need not be linear-in-momentum strictly. We repeat that it is essential for a Fermi surface to enclose a Weyl cone. In the 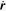 equation, both the second and third terms are anomalous velocity terms. In particular, the second term results in the anomalous Hall effect given by the Berry curvature [29, 30] and the third term gives rise to the chiral magnetic effect [31–36]. The last term in the 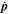 equation is the source of the chiral anomaly, responsible for the negative magneto-resistivity [14, 20, 21, 28]. In this paper, we focus on the electrical-magneto resistivity.

Introducing the topological Drude model into the Boltzmann-equation framework, we arrive at the following expression:

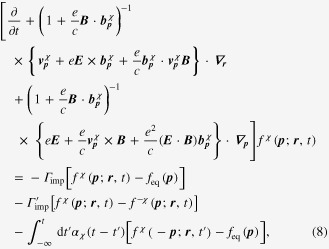
where we introduced an inter Weyl-point scattering term into the Boltzmann equation phenomenologically with the relaxation rate 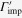 for the inter-node scattering. The weak antilocalization kernel is given by

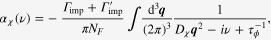
where 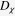 is the diffusion coefficient for each Weyl point, assumed to be identical, i.e., 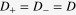.

It is also not very difficult to solve these coupled Boltzmann equations with the generalized Drude model. It follows that electrical transport coefficients can be found from

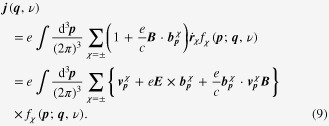
We would like to emphasize that the topological structure of both the Berry curvature and the chiral anomaly modifies the definition of the electric current considerably, which turns out to be essential for anomalous transport phenomena in Weyl metal.

### 2.3. Chiral anomaly in Weyl metal

The next step is to derive the hydrodynamic equation from the Boltzmann equation, performing the coarse graining procedure in momentum space. In normal metals described by the Boltzmann equation (equation (2)) with the conventional Drude model (equation (1)), we find the current conservation law, given by

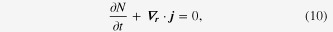
where 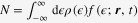 is an electron density with the density of states 
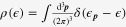
 and 
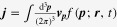
 is an electric current, contributed to dominantly from electrons near the Fermi surface. We recall that collision terms cannot play the role of either sources or sinks for currents [25].

However, the current conservation law breaks down around each chiral Fermi surface [14], considered to be a gauge anomaly [15]. Multiplying 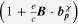 of the Boltzmann equation (equation (8)) with the topological Drude model (equation (7)), we obtain [28]

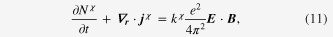
where 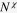 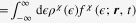 is an electron density with 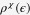 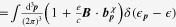 and 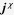 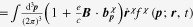 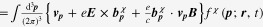 is an electric current around each chiral Fermi surface. 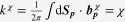 is a magnetic charge at each Weyl point. It is clear that the breakdown of the current conservation law around each Weyl point results from the 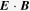 term introduced by the modification of the Drude model. This gauge anomaly around each Weyl point becomes canceled by the existence of its partner. The + chiral charge plays the role of a source in this hydrodynamic equation, whereas the − chiral charge plays the role of a sink. As a result, the total current is conserved, given by

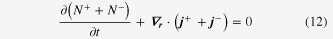
whereas the chiral current is not, described by

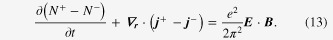
This is the chiral anomaly in three dimensions.

## 3. Anomalous transport phenomena in Weyl metal

An effective Boltzmann transport theory has been constructed for a pair of chiral Fermi surfaces, generalizing the Drude model to incorporate both the Berry curvature and the chiral anomaly. It is straightforward to solve the pair of Boltzmann equations. Here, we do not present details of the calculations in the Boltzmann equation approach. Instead, we focus on novel physics arising from the chiral anomaly. We refer all details of the Boltzmann equation approach to [21].

### 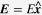 and 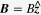


3.1.

In the transverse setup of 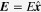 with 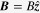, the electrical-magneto conductivity is given by

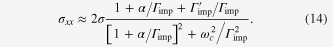
Here 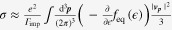 is the Drude conductivity for each Weyl cone, and 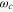 is the cyclotron frequency of the Weyl electron on the chiral Fermi surface. 2 comes from the pair of Weyl cones. 
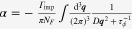
 is the kernel of weak antilocalization in the dc limit. This expression reads

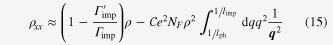
in the leading order for magnetic fields. 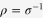 is a residual resistivity due to elastic impurity scattering, and 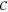 is a positive numerical constant. *l*_imp_ in the upper cut off is the mean-free path, and *l*_ph_ is the phase-coherence length. If one sets 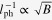 in the lower cutoff, we reproduce the magneto-resistivity with weak antilocalization in three dimensions [37].

The Hall conductivity is

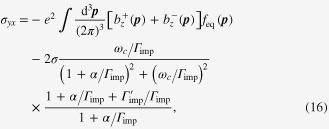
where the first term is an anomalous contribution resulting from the Berry curvature and the second is the normal Hall contribution from the pair of Fermi surfaces with weak antilocalization. Inserting 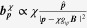 with 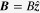 and 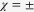 into the expression of the anomalous Hall coefficient and performing the momentum integration, we find that the Hall conductivity is proportional to the momentum-space distance between the pair of Weyl points [21], i.e., 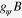, consistent with that based on diagrammatic analysis [29, 30]. For the normal contribution, the presence of the inter-node scattering modifies the Hall coefficient as follows:

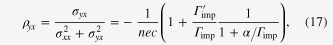

which turns out to be not a constant but a function of magnetic field, combined with the weak antilocalization correction.

### 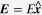 and 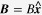


3.2.

In the longitudinal setup of 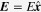 with 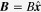, we obtain the longitudinal electrical-magneto conductivity given by

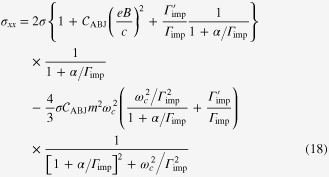
with

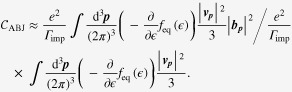


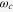
 is the ‘cyclotron’ frequency for the magnetic field in the *x*-direction. If we take the limit of 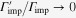, this expression is further simplified as

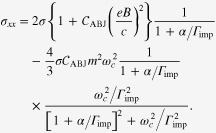
Focusing on the low-field region, we obtain

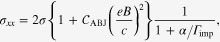
referred to as the enhancement of the longitudinal magneto-conductivity, where the *B*^2^ contribution results from the 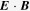 term [14, 20, 21, 28]. Inserting the weak antilocalization correction into the preceding formula and considering 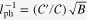 with a positive constant 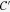, we find

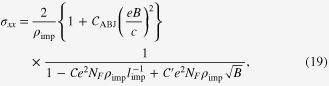
which turns out to fit the experimental data well [20].

It is not that difficult to understand intuitively why the chiral anomaly enhances the longitudinal conductivity. The chiral anomaly allows the dissipationless current channel between the pair of Weyl points, reducing electrical resistivity along the direction of the applied magnetic field [14]. Such an effect appears to be the *B*^2^ term in front of the ‘normal’ resistivity.

Following the same strategy as that of the magneto-conductivity, it is straightforward to find the Hall conductivity around each Weyl point, which consists of anomalous contributions. One is the anomalous Hall effect resulting from the Berry curvature as in the first term of equation (16) but with 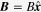, whereas the other is another type of the anomalous Hall effect originating from the chiral anomaly, where the topological 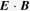 term gives rise to an additional force beyond the conventional Lorentz force. However, we find that the anomaly-induced anomalous Hall effect does not exist, by inserting 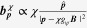 with 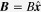 into the expression and performing the momentum integration. The ‘conventional’ anomalous Hall effect also vanishes. In other words, the Hall coefficient turns out to vanish identically in this longitudinal setup [21].

### 3.3. Comparison with experiments

**Figure 2.**
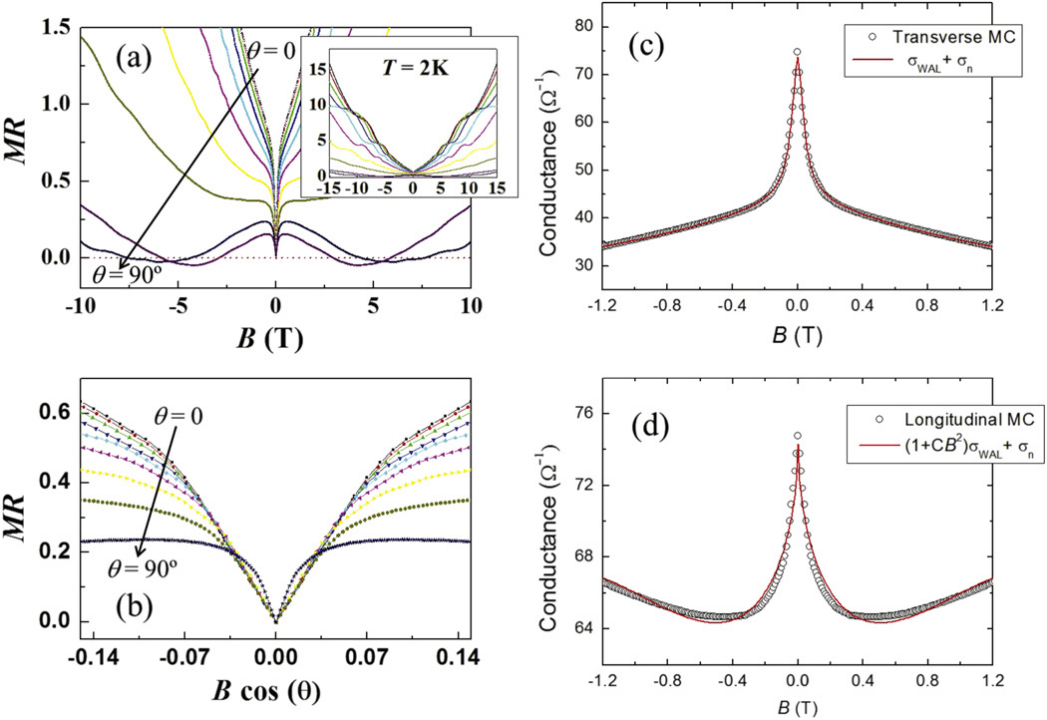
(a) Angle dependence of electrical-magneto resistivity for Bi
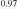
Sb
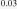
single crystals. 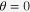 corresponds to the transverse setup of 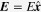 with 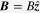, whereas 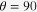 corresponds to the longitudinal setup of 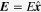 with 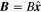. Electrical resistivity becomes suppressed dramatically in the longitudinal setup, resulting from the chiral anomaly between the pair of chiral Fermi surfaces. (b) Three-dimensional weak antilocalization. Breakdown of scaling for the magnetic field of the 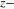 direction implies that this weak antilocalization appears from not the surface state but the bulk state. Indeed, 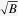 fits this data quite well. (c) Fitting of the transverse electrical-magneto conductivity based on equation (19) without the *B*^2^ term and (d) fitting of the longitudinal electrical-magneto conductivity based on equation (19) with the *B*^2^ term. We also introduced the normal contribution from electrons of the Fermi surface near the 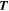 point. For details, see the text. (Figure from Kim *et al* [20].)

To explain the experimental data of [20], we introduced two contributions for magneto-conductivity, where one results from Weyl electrons near the 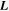 point of the momentum space and the other comes from normal electrons near the 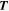 point. Subtracting out the cyclotron contribution of normal electrons in the transverse setup (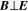), we can fit the data based on the three-dimensional weak-antilocalization formula given by Weyl electrons, where the weak-antilocalization correction has been Taylor-expanded for the weak-field region below 1.2 T. On the other hand, the cyclotron contribution around the 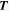 point almost vanishes for the longitudinal setup (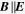) as must be, and the residual resistivity for normal electrons is almost identical with that of the transverse setup. Subtracting out the 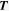 point contribution, we can fit the data with equation (19) in the regime of the weak magnetic field below 1.2 T, where the weak-antilocalization correction has been also Taylor-expanded. Again, the weak-antilocalization correction turns out to be almost identical with that of the transverse setup, whereas we have an additional constant 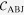 in the longitudinal setup, the origin of which is the chiral anomaly. See figure 2.

**Figure 3.**
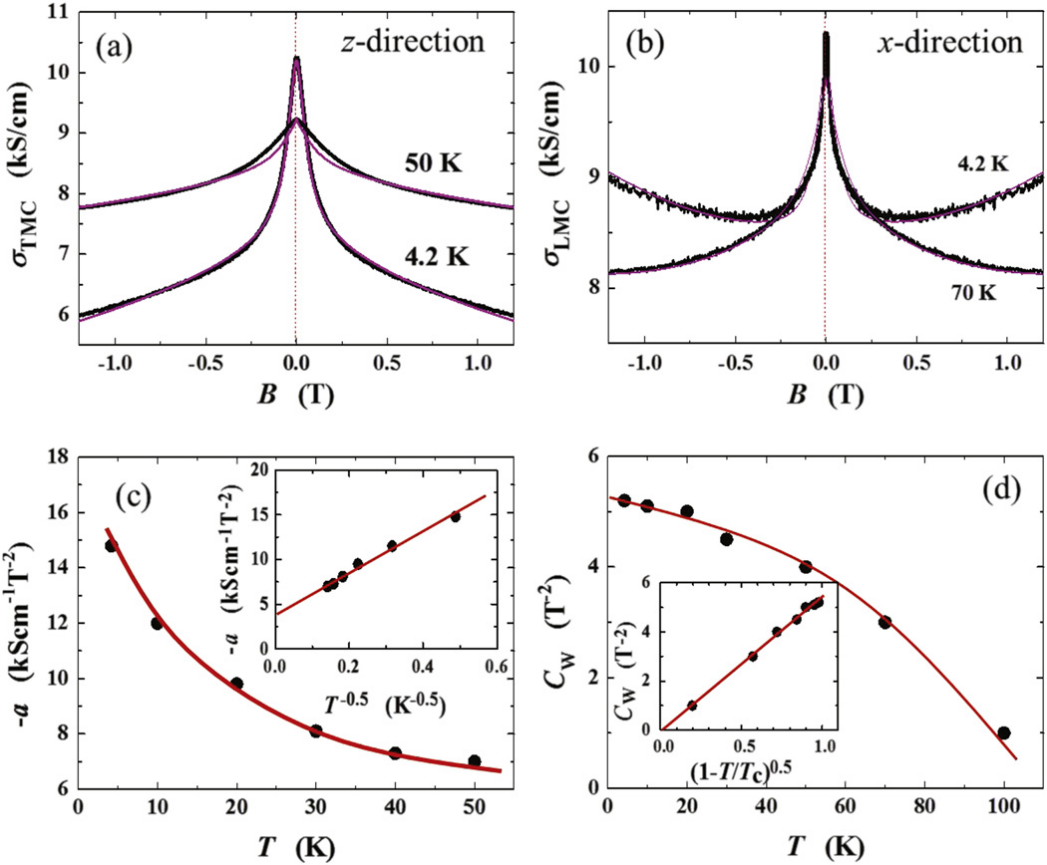
(a) Temperature dependence of transverse electrical-magneto conductivity (TMC: 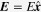 and 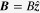) for Bi
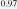
Sb
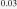
single crystals, well fitted by 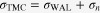 with 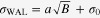 and 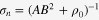, as discussed in the text. The weak anti-localization (WAL) contribution observed in 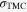 is reduced gradually by increasing the temperature, wherein its magnitude is reduced about 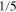 at 50 K compared with that at 4.2 K. (b) Temperature dependence of longitudinal electrical-magneto conductivity (LMC: 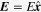 and 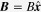) for Bi
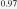
Sb
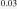
single crystals, well fitted by 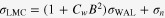. We would like to point out that the weak anti-localization contribution remains more robust than that of TMC, expected to result from the absence of an orbital effect in the longitudinal setup. (c) Temperature dependence of the weak anti-localization contribution in TMC. The coefficient *a* is found to be proportional to 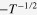, which completes the scaling relation of 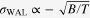 in the three-dimensional weak anti-localization correction. (d) Temperature dependence of the enhancement factor of the *B*^2^ term *C*_*w*_ in LMC. It is quite unexpected that the enhancement factor follows the standard mean-field behavior like an order parameter, i.e., 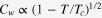 with 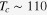 K. These measurements were performed at the Wuhan National High Magnetic Field Center.

Recently we investigated effects of thermal fluctuations on this chiral-anomaly driven enhancement of the longitudinal electrical-magneto conductivity, where temperature dependence of the *B*^2^-enhancement factor *C*_*w*_ has been measured. See figure 3. First of all, we found that the temperature dependence of the enhancement factor follows the standard mean-field behavior like an order parameter, i.e., 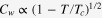 with 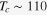 K, which was quite unexpected. We believe that this measurement proposes the interesting question of how thermal fluctuations can affect some topological properties—not much discussed until now.

## 4. How to describe a Weyl metallic state II: topological Fermi-liquid theory

### 4.1. An effective field theory for a pair of chiral Fermi surfaces

#### 4.1.1. First quantization: derivation of the topological Drude model

To construct a topological Fermi-liquid theory, we start from the dynamics of one Weyl electron. The time evolution operator is given by

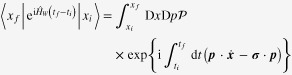
in the path integral representation. 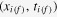 denotes an initial (final) point in the configuration space, and 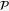 means ‘path ordering’. The Weyl Hamiltonian is given by 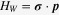 for a Weyl point with + chirality, where 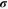 is a Pauli matrix representing spin.

We focus on the case when the chemical potential lies away from the Weyl point, allowing a Fermi surface. We derive an effective Hamiltonian for the dynamics of such a Weyl electron on the Fermi surface. An idea is to reformulate the Weyl Hamiltonian as follows, resorting to the CP^1^ representation [33]:

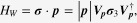
where 
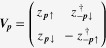
 is a two-by-two matrix with a unimodular constraint of 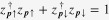. This is nothing but a change of basis, which diagonalizes the Weyl Hamiltonian. The path integral representation becomes also reformulated as follows [33]:

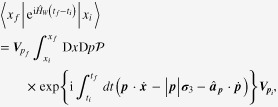
where 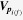 is an SU(2) matrix at an initial (final) position. As clearly seen, we have a Berry phase term within this diagonal basis:

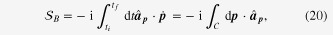
where 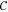 is a path of a Weyl electron adiabatically moving on the Fermi surface and

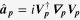
is an SU(2) Berry gauge field.

Introducing electromagnetic fields into the Weyl Lagrangian and reformulating it based on the diagonalized basis more carefully, one finds an effective action that describes the dynamics of a Weyl electron on the Fermi surface [38–40]:

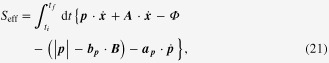
where 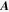 and *Φ* are the electromagnetic vector potential and scalar potential, respectively, and the 11 component of the SU(2) Berry gauge field is chosen to be

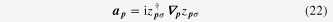
and 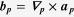 is the Berry curvature field near the Fermi surface.

It is straightforward to find a set of semi-classical equations of motion, given by [32, 33]:

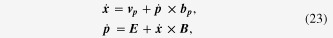
where 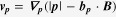 is the group velocity, renormalized by the relativistic correction of the magnetic-moment coupling. This is the topological Drude model that we discussed in section 2. The solution is

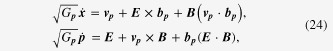
where 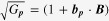 is a measure of the phase space with chiral charge.

#### 4.1.2. Second quantization: toward a topological Fermi-liquid theory

The next step is to reformulate the first-quantized effective Weyl action in the second quantization, which describes the dynamics of many electrons near the Fermi surface enclosing a Weyl point. An idea is to introduce a spinless fermion field at the chiral Fermi surface, related to an original Weyl electron via the CP^1^ representation as follows:

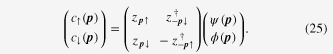
Because we focus on low-energy dynamics near the chiral Fermi surface, we find the relation between the spinless fermion field and the original Weyl electron field, given by

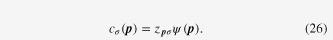



Resorting to this slave-fermion representation for Weyl metal, we can reformulate the gauge-matter coupling term in the imaginary-time formulation at a finite temperature as follows:

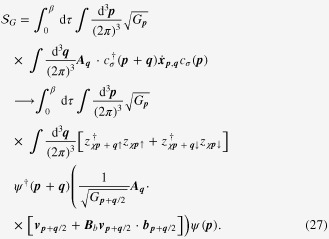
Here the solution to the semi-classical equation of motion has been introduced explicitly, where a constant background magnetic field 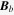 is applied for a Weyl metallic state but without an electric field. The factor of 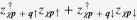 counts the reduction of an overlap between wave functions at different momentum points, which originates from the spin-momentum locking. The gauge-matter coupling vertex becomes modified considerably according to the generalized Drude model for the chiral Fermi surface, where the chiral anomaly generates anomalous velocity terms. In the same way, we are allowed to rewrite the Berry-phase term as follows:

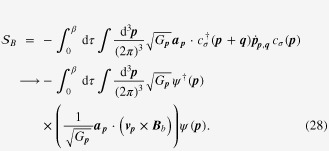



The preceding discussion leads us to propose an effective field theory for a Weyl metallic phase:

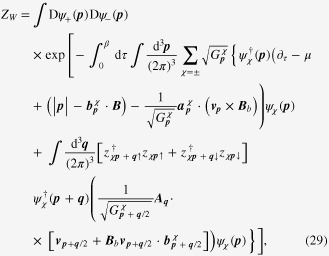
where a pair of chiral Fermi surfaces has been marked explicitly with *χ*. The linear dispersion of a Weyl electron on the chiral Fermi surface gets a correction from the Berry-phase term. The electric current coupled with the electromagnetic gauge field also becomes generalized due to the chiral anomaly.

### 4.2. Enhancement of the longitudinal electrical-magneto conductivity within the Kubo formula

One can derive the longitudinal positive electrical-magneto conductivity within the Kubo formula based on the effective field theory for a pair of chiral Fermi surfaces. Taking derivatives of the free energy 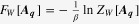 twice with respect to the electromagnetic vector potential 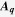, we obtain the following expression for the current–current correlation function:

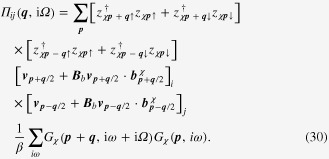
Here,

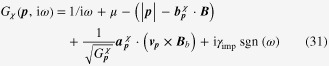
is the propagator of the spinless fermion near the chiral Fermi surface, where the contribution of impurity scattering has been introduced within the Born approximation [37]. 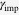 is a scattering rate of the spinless quasiparticle.

Taking the limit of 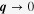 in this polarization function, we obtain the dc conductivity

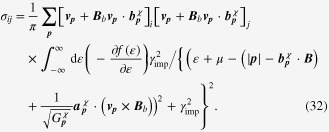
It is straightforward to perform the momentum integral. As a result, we recover the enhancement of the longitudinal electrical-magneto conductivity of the Boltzmann-equation approach with the topological Drude model [14, 20, 21, 28]

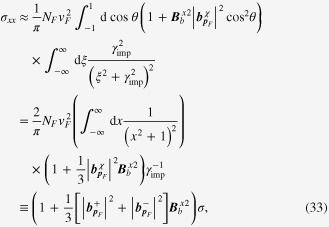
where *σ* is the residual conductivity determined by the impurity scattering rate 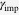, the Fermi velocity *v*_*F*_, and the density of states at the Fermi energy *N*_*F*_.

## 5. Summary and discussion

In this review article, we have discussed two kinds of theoretical frameworks for anomalous transport phenomena in Weyl metal, where one is the Boltzmann-equation approach and the other is an effective field-theory approach. An essential point was how to encode the topological structure of the Weyl metallic state given by both the Berry curvature and the chiral anomaly. In both theoretical frameworks, the idea was to generalize the Drude model for the dynamics of the Weyl electron on the chiral Fermi surface. Resorting to the topological Drude model, we could construct not only the Boltzmann-equation approach but also a topological Fermi-liquid field theory for the topological metallic phase with a pair of chiral Fermi surfaces. Both approaches gave rise to the enhancement of longitudinal electrical-magneto conductivity consistently.

### 5.1. Derivation of the Boltzmann equation with the topological Drude model

We would like to emphasize that the current phenomenological Boltzmann-equation approach is applicable only when the chemical potential lies away from the Weyl point, forming a pair of chiral Fermi surfaces. When the chemical potential touches the Weyl point, we should re-derive the Boltzmann equation from the effective field theory for quantum electrodynamics in one-time and three-space dimensions (QED_4_) under the magnetic field. The distribution function in this relativistic Boltzmann equation will be expressed as a 4 × 4 matrix because the lesser Greenʼs function consists of the four-component spinor. An interesting and fundamental problem is as follows. Taking the non-relativistic limit from the matrix Boltzmann equation when the chemical potential lies above the Weyl point, can we reproduce the current Boltzmann-equation framework, where effects of other components except for the Fermi-surface component are ‘integrated out’ or ‘coarse grained’, giving rise to such contributions as semi-classical equations of motion? This research will give a formal basis to the current phenomenological Boltzmann-equation approach. Recently, the Boltzmann-equation framework was derived from QED_4_, based on the introduction of the Wigner function to satisfy a quantum kinetic equation [38, 41, 42]. These derivations imply that Lorentz symmetry, gauge symmetry, and quantum mechanics are important ingredients for the existence of the chiral anomaly.

### 5.2. Derivation of the topological Fermi-liquid theory

We would like to point out that the standard diagrammatic approach based on QED_4_ fails to incorporate contributions from the chiral anomaly for some physical quantities. Although the diagrammatic approach based on QED_4_ with a finite chemical potential succeeds in calculating the anomalous Hall effect [29, 30] and the chiral magnetic effect [35, 36], both of which are associated with transverse contributions of the current–current correlation function, it fails to describe the negative longitudinal magneto-resistivity associated with the longitudinal component of the current–current correlation function. An effective action for single-particle dynamics has been constructed in the phase space, imposing the momentum-space Berry gauge field. Reformulating the Berry phase based on Stokeʼs theorem, a five-dimensional gauged Wess–Zumino–Witten term has been proposed to reproduce chiral anomalies associated with not only electromagnetic but also gravitational fields and their mixing [43]. If this action functional is reformulated in the second quantized form, an effective field theory for the topological Fermi-liquid state will be given by Landauʼs Fermi-liquid theory backup with the five-dimensional gauged Wess–Zumino–Witten term. Recall the problem of a spin chain, where the spin 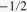 chain is described by the O(3) nonlinear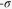 model backup with a Berry phase term, whereas such a topological term is irrelevant for the spin-1 chain with a periodic boundary condition [44]. Unfortunately, this fascinating construction does not seem to be very practical for actual calculations because it is not easy to introduce the role of such a term into diagrammatic analysis as in the case of field theories with topological terms.

On the other hand, we have introduced the role of both the Berry curvature and the chiral anomaly within the U(1) slave-fermion representation. The orthogonality of the wave-function overlap and the Berry gauge field in momentum space are expressed in terms of the CP^1^ bosonic field whereas the dynamics of the pair of chiral Fermi surfaces are described by the spinless fermion field, where the chiral anomaly plays the role of an effective potential, modifying the dispersion of the Weyl electron and the gauge coupling with the spinless fermion field. This phenomenological construction should be derived from QED_4_ under magnetic fields, where high-energy fluctuations except for low-energy modes near the Fermi surface are ‘integrated out’ or ‘coarse grained’, expected to cause anomaly-induced effective potentials as suggested in this manuscript [22]. We believe that this way serves as a novel direction, deriving the chiral anomaly in the presence of a Fermi surface.

### 5.3. Thermal transport and violation of the Wiedemann–Franz law

Recently one of the authors investigated thermal transport coefficients based on the Boltzmann transport theory with the topological Drude model [23]. We found that not only the longitudinal electrical-magneto conductivity but also both the Seebeck and thermal conductivities in the longitudinal setup show essentially the same enhancement proportional to *B*^2^, the origin of which is the chiral anomaly. Actually, our preliminary experimental result regarding longitudinal thermal conductivity seems to show behavior similar to that of longitudinal electrical-magneto conductivity [45]. An interesting prediction of this theoretical study is that the Wiedemann–Franz law will be violated only in the longitudinal setup, showing the *B*^2^ dependence on the Lorentz number. On the other hand, the Wiedemann–Franz law turns out to hold in the transverse setup as expected. This prediction is quite surprising because the concept of an electron quasiparticle is still valid. The origin of the violation is that the coefficient in front of *B*^2^ differs from each transport coefficient, i.e., 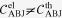, where the superscript of el (th) means electrical (thermal). Because the breakdown of the Wiedemann–Franz law appears from this term, the chiral anomaly is the mechanism, implying that this metallic state should be distinguished from Landauʼs Fermi-liquid state.

### 5.4. The connection between the chiral anomaly and the surface Fermi-arc state

In this paper, we have focused on bulk transport because we were considering a metallic phase with a pair of Fermi surfaces. However, the emergence of the surface Fermi-arc state is regarded as the hallmark of the Weyl metallic state [10], exactly analogous to the Weyl point on the surface state of a topological insulator, where each Fermi point of the Fermi arc corresponds to the Weyl point in the case of the topological insulator. The standard argument for the existence of the surface Fermi-arc state is as follows [10]. Assume a pair of Weyl points in the *z*-direction, given by 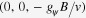 and 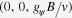, respectively. If we fix the momentum of 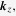 we are in the two-dimensional plane, allowing us to define the Chern number, which counts the number of magnetic monopoles in momentum space. Considering the two-dimensional plane at 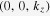 with 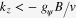, we see that the Chern number vanishes. However, considering two dimensional planes with 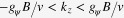, we find that the Chern number is 1 because such two-dimensional planes cross the Weyl point. If the plane lies in 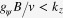, it crosses both the + and − charges, causing the Chern number to vanish. Because the two-dimensional planes with the Chern number 1 are essentially the same as the integer quantum Hall state, we have an edge state for each *k*_*z*_ within 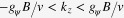, giving rise to a Fermi arc state on the surface. Unfortunately, this spectroscopic fingerprint has not been observed yet.

In our opinion, the connection between the chiral anomaly and the surface Fermi-arc state has not been clarified yet. Although the Hall current through the bulk channel can be derived from the topological-in-origin 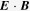 term [10], the connection between the bulk Hall current and the surface Hall current is not discussed completely as far as we understand. We believe that this issue should be clarified further.

## Appendix. What makes Weyl metal special: axion electrodynamics

The effective Dirac Hamiltonian of    in momentum space gives its corresponding effective action of    in real space. Representing two kinds of Pauli matrices in terms of Dirac gamma matrices of  and , we are allowed to rewrite this expression as the standard representation of the Dirac theory, given by   , where  is a conjugate partner of .

Introducing electromagnetic fields into this expression, we obtain QED_4_ (quantum electrodynamics in one time and three spatial dimensions):

where the topological-in-origin  term has been incorporated.  is the field strength tensor, and  recovers Maxwell equations through the variational principle.  is an antisymmetric tensor, and  is nothing but the  term.

Replacing the topological-in-origin term with a chiral current based on the anomaly equation

one may rewrite the preceding expression as follows:

where the chiral gauge field  appears through the technique of the integration by part, given by .

It is quite interesting to observe that the Zeeman energy term plays essentially the same role as the chiral gauge field, given by

where the chiral gauge field is identified by  and . This indicates that the axion angle is given by .

It is straightforward to integrate over gapped fermion excitations, resulting in an effective field theory for electromagnetic fields:

where time dependence in  has been introduced for generality. Applying the least-action principle to this effective Lagrangian, we arrive at Maxwell equations to describe the axion electrodynamics:

where we follow the standard cgs notation with  and the fine structure constant *α* [24].
